# *Yersinia pseudotuberculosis* in Non-Domesticated Mammals and Birds in Captivity

**DOI:** 10.3390/vetsci12020161

**Published:** 2025-02-12

**Authors:** Remco A. Nederlof, Linda G. R. Bruins-van Sonsbeek, Job B. G. Stumpel, Hester van Bolhuis, Els M. Broens, Jooske IJzer, Jaco Bakker

**Affiliations:** 1 Independent Researcher, 2861 XZ Bergambacht, The Netherlands; 2 Anatomy and Physiology Section, Department Clinical Sciences, Veterinary Faculty, Utrecht University, Yalelaan 1, 3584 CM Utrecht, The Netherlands; g.r.vansonsbeek@uu.nl; 3 WILDLANDS Adventure Zoo Emmen, Raadhuisplein 99, 7811 AP Emmen, The Netherlands; j.stumpel@wildlands.nl; 4 AAP, Animal Advocacy and Protection, Kemphaanpad 1, 1358 AC Almere, The Netherlands; hester.van.bolhuis@aap.nl; 5 Veterinary Microbiological Diagnostic Centre, Department Biomolecular Health Sciences, Veterinary Faculty, Utrecht University, Yalelaan 1, 3584 CM Utrecht, The Netherlands; e.m.broens@uu.nl; 6 Division of Pathology, Department Biomolecular Health Sciences, Veterinary Faculty, Utrecht University, Yalelaan 1, 3584 CM Utrecht, The Netherlands; j.ijzer@uu.nl; 7 Animal Science Department, Biomedical Primate Research Centre, Lange Kleiweg 161, 2288 GJ Rijswijk, The Netherlands; bakker@bprc.nl

**Keywords:** *Yersinia pseudotuberculosis*, yersiniosis, zoo, captivity, transmission, mortality, diagnosis, pathology, vaccine, treatment

## Abstract

*Yersinia pseudotuberculosis* causes yersiniosis in a wide range of mammalian and avian taxa worldwide. This review aims to provide an overview of the current literature on yersiniosis in non-domesticated mammals and birds in captivity. Data on the prevalence of *Y. pseudotuberculosis* in captive populations are scarce. Transmission is primarily via the fecal–oral route, with wild rodents and birds as primary reservoirs. Predisposing factors to yersiniosis include young age, inclement weather, stress, and genetic factors. Symptoms are often non-specific, with sudden death occurring frequently, particularly in avian species. Gross pathological examination typically reveals multiple white-yellow foci in visceral organs, while histopathology demonstrates necrosis with central bacterial colonies. Ante-mortem diagnosis can be challenging as bacterial culture and isolation from fecal samples are often hindered by intermittent excretion and competition with other gastrointestinal flora. Although killed and subunit vaccine formulations have demonstrated limited protective efficacy, live attenuated strains may hold greater promise for inducing more robust and durable immunity. *Y. pseudotuberculosis* remains a significant threat to animal health, highlighting the need for faster and more reliable diagnostic techniques and the development of more effective treatment and prophylactic strategies.

## Simple Summary

*Yersinia pseudotuberculosis* is a zoonotic bacterial pathogen that causes yersiniosis in a wide range of mammals and birds, both in the wild and in zoo animals around the world. This bacterium is transmitted via the fecal–oral route, with wild rodents and birds serving as reservoirs. Symptoms are often non-specific, with sudden death being a common manifestation of the disease. Pathological examination typically reveals multiple white-yellow foci in visceral organs, while microscopy shows necrosis with central bacterial colonies. The diagnosis in live animals can be challenging. Once animals become symptomatic, treatment is often ineffective. Therefore, emphasis should be placed on identifying risk factors for yersiniosis and developing evidence-based biosecurity and prophylactic strategies. Although killed and subunit vaccines have demonstrated limited protective efficacy, live attenuated vaccines may constitute viable alternatives.

## 1. Introduction

*Yersinia pseudotuberculosis* is a small, Gram-negative, and facultatively anaerobic pleomorphic coccobacillus and the causative agent of clinical yersiniosis. *Y. pseudotuberculosis* has been classified into 21 serotypes based on the O-antigen, of which only some serotypes are considered to be pathogenic [1]. Serotype O:1 has been observed to be the most common serogroup in Europe, has been isolated from wild swine, rodents, and bird species, and has been implicated in infections in zoological settings [2,3,4,5,6]. Outside of Europe, other serotypes have been reported to cause disease in wild animals and in zoological settings [7]. In addition, serotype 3 was isolated in various deer species in the United States (*n* = 25) [8], and serotypes 1b, 2b, 3, 4b, 6, and 7 have been isolated from deceased monkeys of various species in Japan [9,10]. Avirulent strains of *Y. pseudotuberculosis* have been identified and are present in wild animals and the environment [11]. 

A multitude of virulence factors are described, some of which are shared with other *Yersinia* spp. [12]. Virulence factors may be encoded chromosomally or on a virulence plasmid. The functions of these virulence plasmids include iron uptake, facilitating adhesion to and entry into host cells, and modulation of host cell signaling pathways. The presence of a 70 kb virulence plasmid (pYV) is associated with increased pathogenicity. Both highly pathogenic pYV-positive bacteria and less pathogenic pYV-negative bacteria may exist in the same serogroup [3,11]. The pYV encodes for a type-III secretion system (T3SS), *Yersinia* outer membrane proteins (Yops), as well as *Yersinia* adhesin A (YadA). Yops are secreted proteins that, when delivered into the cytosol of the target host cells by means of the type-III secretion system, interfere with signaling pathways for phagocytosis, apoptosis, inflammatory response, actin cytoskeleton regulation, and cytokine production and may inhibit phagocytosis [13,14]. YadA, a virulence plasmid-encoded protein, is the major adhesin responsible for *Yersinia* contact with cells after crossing the intestinal epithelium [15].

*Yersinia pseudotuberculosis* is considered to be zoonotic and can cause disease in a wide range of mammalian and avian taxa [2,16,17]. *Y. pseudotuberculosis* is often implicated in lethal epizootics in zoo animals, but individual cases have also been described [18]. It is transmitted via the fecal–oral route, and wild rodents and birds are considered reservoirs [3,7,16,19]. However, the bacteria may persist in the environment for a long time as *Y. pseudotuberculosis* is a psychrotrophic bacterium capable of growth at temperatures between 4 and 20 °C [20]. This has resulted in concerns regarding reservoir formation and the occurrence of new epizootics. As a result, zoological institutions must remain vigilant to protect the health of the animals in their collections, as well as the health of staff and the general public.

This review aims to summarize the current understanding of the occurrence, ante-mortem and post-mortem characteristics, diagnostic methods, treatment options, and prophylactic measures for *Y. pseudotuberculosis* in non-domesticated mammals and birds in captivity. It highlights knowledge gaps and research opportunities and aims to provide veterinary and animal management staff with relevant information for the management of *Y. pseudotuberculosis* in a zoological setting. To identify the relevant literature, we conducted a search for conference abstracts, peer-reviewed publications, newsletters, books, and book chapters in academic literature databases, including PubMed, Scopus, and Google Scholar.

## 2. Transmission

*Yersinia pseudotuberculosis* is transmitted via the fecal–oral route by ingestion of food or water contaminated with feces from natural reservoir species, mainly birds and rodents [3,7,16,19]. A study performed in Britain isolated *Y. pseudotuberculosis* from 21 native bird species and five native rodent species [21]. Additionally, a study on migratory bird species in Sweden isolated *Y. pseudotuberculosis* in 0.6% of caught birds, including two song thrushes (*Turdus philomelos*) and a redwing thrush (*Turdus iliacus*), with all birds carrying pathogenic strains of *Y. pseudotuberculosis* [22]. A survey in Japan in the 1990s isolated *Y. pseudotuberculosis* from 2.9% (44/1530) of wild mice [23], and a recent survey carried out in southeastern Poland isolated *Y. pseudotuberculosis* from 0.5% (1/214) of striped field mice (*Apodemus agrarius*) [24]. A survey in Finland isolated serotype O:2 *Y. pseudotuberculosis* from 0.7% of common shrews (*Sorex araneus*) (2/293) but failed to isolate the pathogen in voles (*n* = 117) and mice (*n* = 376) [25]. Moreover, it has been proposed that cattle served as a reservoir for a serotype O:3 *Y. pseudotuberculosis* strain that was isolated from deer in the United States [8,26]. Infections have also been reported in wild carnivores and omnivores, suggesting the additional possibility of transmission through the consumption of infected prey species [2,19]. Although studies from various geographic regions indicate a consistently low prevalence of *Y. pseudotuberculosis* among carrier species, the overall data are sparse, highlighting the need for more comprehensive surveillance efforts. The relative role of avian versus rodent reservoirs in yersiniosis outbreaks is poorly understood and may change depending on geographical and institution-specific risk factors. Furthermore, the role of conspecifics as asymptomatic carriers is not well understood and may constitute an additional route of transmission in zoological institutions. 

The presence of *Y. pseudotuberculosis* has been demonstrated in water and soil and on vegetation, providing additional routes of infection [2,3]. Moreover, *Y. pseudotuberculosis* may persist in the environment for a long time as it is considered to be psychrotrophic. The bacterium is capable of growth at temperatures between 4 and 20 °C and is able to survive temperatures as low as −20 °C [20]. Consequently, while the role of fomite transmission has not been extensively studied, it should not be discounted. It has, however, been suggested that *Y. pseudotuberculosis* may lose its virulence genes during adaptation to a saprophytic lifestyle in the soil. This may reduce the risk of reinfection from the soil in places where infection occurred over nine months ago [27].

## 3. Occurrence and Risk Factors

### 3.1. Incidence and Prevalence

Unfortunately, most evidence for yersiniosis is obtained during post-mortem examination (see Section 5). Incidence and prevalence data are therefore largely unavailable, except for some reservoir species (see Section 2).

The high population density of nonhuman primates (NHPs) housed in zoological institutions and research centers increases the risk of *Yersinia* sp. infection. One survey in three closed research colonies (*n* = 61, 49, and 41 animals) of male rhesus macaques (*Macaca mulatta*) failed to isolate enteric *Y. pseudotuberculosis* and *Y. enterocolitica* from deep rectal swabs obtained during routine health evaluation. Among the isolates, only one species of *Yersinia*, namely, *Y. ruckeri*, was identified in 6.0% (9/150) of the samples. However, this species is not considered to be pathogenic in mammals. Although not detailed, we assume these monkeys were housed indoors as the monkeys were participants in a renal transplant immunology project [28].

In other mammalian and avian species, no information is available on the prevalence of *Y. pseudotuberculosis* in healthy animals. Moreover, no data exist on the incidence of non-lethal yersiniosis in zoological species. As a result, the clinical significance of incidental *Y. pseudotuberculosis* cultures in healthy animals remains unknown. Future research may address this gap in knowledge by investigating the occurrence of *Y. pseudotuberculosis* in healthy birds and mammals in captivity. 

### 3.2. Risk Factors

In zoological institutions, the disease has been mainly reported in bovids, cervids, felids, bats, NHPs, rodents, hornbills, toucans, pigeons, and parrots (see Appendix A, Table A1 and Table A2) [6,7,29]. Additionally, domestic pet species and farm animal species also appear to be susceptible [18,30]. 

Across taxa, clinical yersiniosis seems to occur more frequently in juvenile animals [29,31]. In some instances, animals were only a few months old at the time of infection [8,16,32]. It has been proposed that immaturity of the immune system may heighten susceptibility to infection, which is reflected in the frequently reported young age of animals afflicted by *Y. pseudotuberculosis* (see Appendix A, Table A1 and Table A2) [32]. Moreover, stress surrounding the time of weaning may also predispose to infection [8].

Stress is proposed to be a predisposing factor as it suppresses protective immune responses [33]. A small outbreak has been reported following a period of stress associated with increased ambient noise and activity in primates [29]. A small increase surrounding a birth peak in cervids and bats may imply that peripartum stress is a predisposing factor to the development of yersiniosis [8,16]. Other stressors, e.g., concurrent disease or social stress, may also play a role. Outbreaks of yersiniosis have occurred in captive populations of Egyptian rousette bats (*Rousettus aegyptiacus*) living in an indoor climate-controlled environment during the summer. In this case, it was considered that social stress resulting from overpopulation predisposed animals to disease [31].

Most cases are described to occur in the autumn and winter [8,16,29,32,34,35,36,37]. Unfavorable weather conditions, particularly exposure to cold and rainy weather, are suggested to increase susceptibility to infection [8,32]. Stress resulting from maladaptation to such climates may contribute to this pattern in tropical species. However, it is more likely that this trend reflects the tendency of rodent vectors to seek shelter indoors during the cold season. 

Excessive iron storage in birds, mice, and humans is suggested to predispose to yersiniosis as the excess iron is believed to facilitate the survival and proliferation of *Y. pseudotuberculosis* [5,38,39]. A study conducted in chickens (*Gallus domesticus*) suggests that iron excess facilitates the development of systemic pseudotuberculosis due to increased iron availability [38]. In particular, toucans (*Ramphastidae*), which are prone to developing iron storage disease, are considered to be highly susceptible to the development of pseudotuberculosis [7,35,40]. The role of iron metabolism in the susceptibility to yersiniosis warrants further research, especially since dietary iron availability can be controlled through husbandry practices in zoological collections.

Investigating the relative risk of developing yersiniosis in animals incidentally found to harbor *Y. pseudotuberculosis*, or in negative animals cohabitating with positive animals, compared to negative populations, may provide deeper insights into the risk factors associated with clinical disease.

## 4. Clinical Symptoms

Yersiniosis is characterized by high mortality (see Appendix A, Table A1 and Table A2). However, it is likely that the data presented here overestimate the mortality as subclinical cases may not be diagnosed, animals may recover uneventfully, and data are often not published. 

Yersiniosis in mammals is commonly described as a primary gastrointestinal disease, manifesting as ulcerative enteritis, mesenteric lymphadenomegaly, and hepatitis [32,41]. These lesions are sometimes referred to as type 1 lesions [42]. Yersiniosis in birds is more commonly described as lymphoreticular or type 2, commonly manifesting as hepatitis and splenitis [42]. Nevertheless, both type 1 and type 2 lesions have been described in different taxa. Moreover, both type 1 and type 2 lesions may occur concurrently in the same animal [43].

Clinical symptoms in mammals are often limited to anorexia, hyporexia, lethargy, and sometimes diarrhea. Death without premonitory signs, may, however, also occur (see Appendix A, Table A1). This has been reported in scimitar-horned oryx, axis deer (*Axis axis*), Chinese water deer (*Hydropotes inermis*), Seba’s short-tailed bats (*Carollia perspicillata*), squirrel monkeys (*Saimiri* sp.), Patagonian mara (*Dolichotis salinicola*), and capybaras (*Hydrochoerus hydrochaeris*) (see Appendix A, Table A1).

Neurological symptoms have been reported in mammals, with one silvery marmoset (*Mico argentatus*) developing paraplegia and with ataxia being reported in a gemsbok (*Oryx gazella*), a scimitar-horned oryx (*Oryx dammah*), and silvery marmosets (see Appendix A, Table A1). Ataxia and paraparesis have been reported in a Patagonian mara, but these likely resulted from a chronic subcutaneous phlegmona above the lumbar spine [6].

In cervids, sudden death has most frequently been observed, although diarrhea, emaciation, and dehydration have been observed in affected animals [8]. It appears that sudden death is less common in artiodactyls. Although disease progression is still rapid, mostly within a single day, it has been reported to be as long as 12 days in a scimitar-horned oryx [29].

Jaundice has been observed in a single report about a cougar (*Felis concolor*) with hepatic yersiniosis. This animal also demonstrated anorexia for two weeks prior to death [44]. Dyspnea has been reported in two male lions (*Panthera leo leo*), of which one died after a five-day course of clinical disease [45].

In Seba’s short-tailed bats, a case of abortion has been reported a week prior to death due to *Y. pseudotuberculosis*, suggesting that infection may cause abortion in bats, similar to what has been observed in ewes and goats [16,41,46]. Endometritis was observed in this animal during post-mortem examination [16]. Egyptian fruit bats may exhibit both acute yersiniosis characterized by sepsis and multi-organ failure, as well as chronic yersiniosis, characterized by necrotic abscessation in one or multiple organs, particularly the liver, spleen, and mesenteric lymph nodes [31,47].

In NHP, yersiniosis is described to cause both gastrointestinal and systemic diseases, with potentially lethal outcomes [6,10,29,34,48,49,50,51,52,53,54,55,56,57,58,59,60,61,62,63,64]. It is notable that the manifestation of clinical symptoms preceding mortality can be non-existent or subtle; however, documented clinical presentations include diarrhea (sometimes hemorrhagic), lethargy, anorexia, posterior paralysis, significant weight loss, depression, dehydration, and hypothermia within a 24-h period preceding death. Abdominal distension has been reported as the sole clinical symptom in a white-faced saki (*Pithecia pithecia*), other than unresponsiveness [29]. Erythematous and punctate rashes have been reported in primates, similar to those observed in humans [29]. It has been proposed that the presence of the ypmA gene, encoding for YPM, may be associated with atypical manifestations of disease in primates, such as rash, desquamation, erythema nodosum, and arthritis [9].

In birds, sudden death is most common. Clinical symptoms are often limited to lethargy, diarrhea, and anorexia or hyporexia (see Appendix A, Table A2). Bright green feces have been reported in blue-fronted amazons (*Amazona aestiva*) and yellow-headed amazons (*Amazona oratrix*), which were likely related to anorexia or may have been associated with hepatic changes observed during necropsy. These birds demonstrated lethargy, ruffled feathers, and diarrhea before death [5].

## 5. Post-Mortem Examination

### 5.1. Gross Pathology

Most evidence for yersiniosis is obtained during post-mortem examination. Lesions reported in the literature are summarized in Table A3 and Table A4 (see Appendix B). Reported gross pathological changes depend on varying degrees of interpretation. As a result, the columns of Table A3 and Table A4 may contain terminology with partially overlapping definitions. Based on macroscopic findings, inflammatory lesions were frequently reported in both birds and mammals. However, histological confirmation, or at least cytological indication, is required to determine the presence of inflammation. Nevertheless, inflammation has been incorporated in Table A3 and Table A4 based on the available descriptions in the literature. Thus, terminology such as enteritis and pneumonia in these tables must be read within this limitation. 

#### 5.1.1. Gross Pathology of Mammals

Due to the rapid onset of clinical signs and death, body condition is likely to be unaffected in (per)acute disease [7,65] (see Appendix B, Table A3). 

A common finding in post-mortem examination is the presence of multifocal white-yellow nodules in various organs. These lesions have been reported most frequently in the liver (48.3%; 42/87), spleen (28.7%; 25/87), lungs (19.3%; 17/88), and mesenteric lymph nodes (13.8%; 12/87) and are rarely observed in the intestines (5.7%; 5/87) and kidneys (1.1%; 1/87) (see Appendix B, Table A3). It is important to note that in both mammals and birds, bacteria may be present in tissues without grossly appreciable lesions [44].

Effusion in the pleural and/or peritoneal cavity is infrequently reported in mammals. One instance has been reported in an African lion. However, effusions have been observed in all squirrel monkey and capybara cases (see Appendix B, Table A3).

The most commonly reported lesions of the respiratory system are white-yellow nodules on the lungs, (broncho)pneumonia, pulmonary hyperemia, pulmonary edema, and pulmonary petechiae (see Appendix B, Table A3). Acute fibrinopurulent pneumonia was reported in captive capybaras [32,65], whereas necrosuppurative pneumonia was observed in Seba’s short-tailed bats [16]. In lions, petechial hemorrhages and ecchymoses were observed on the pleural surface, in the lung parenchyma, and in the endocardium [45]. Petechiae and ecchymoses may be perimortem changes but are likely secondary to sepsis and systemic vasculitis.

Lymphadenopathy of the mesenteric lymph nodes is a common finding across taxa, which may or may not be accompanied by the presence of white-yellow nodules on the lymph nodes (see Appendix B, Table A3). 

A combination of multifocal necrotizing splenitis and hepatitis appears to be common in many species [6,7,18]. The spleen is frequently affected, may be enlarged, and may contain white-yellow nodules. It is seldom reported to be hyperemic (see Appendix B, Table A3). The liver is also often affected, may be covered in white-yellow nodules, may be enlarged, and is infrequently reported to be hyperemic (see Appendix B, Table A3).

White-yellow nodules are uncommonly reported to be present on the intestines. Hemorrhages in the gastrointestinal tract are reported infrequently across mammalian taxa (see Appendix B, Table A3). Enteritis is frequently observed in artiodactyls, mostly presenting as a multifocal hemorrhagic enteritis that primarily affects the small intestines, but similar inflammation may occasionally be observed in the stomach and/or large intestine [29]. Edema of the gastrointestinal tract wall has been reported in a Bolivian squirrel monkey (*Saimiri boliviensis*) [10].

Stomatitis and glossitis have been reported in a single Seba’s short-tailed bat, which also demonstrated hepatitis, enterocolitis, splenitis, and lymphadenitis [16].

Renal lesions are infrequently reported in mammals and are limited to renal hyperemia in capybaras, renal petechiae in squirrel monkeys, and white-yellow nodules on the kidneys in an African lion (see Appendix B, Table A3).

Reported cardiac lesions are limited to petechiae and/or ecchymoses on the endocardium of an African lion and subepicardially in squirrel monkeys [45,66].

A single instance of adrenalitis has been reported in a paca (*Cuniculus paca*) [67], and petechial hemorrhages on the skin have only been reported in silvery marmosets (*Mico argentatus*) [29].

Traditionally, lesions have been categorized into types 1 and 2. However, it is evident that pathological changes in both the gastrointestinal and lymphoreticular systems can manifest across mammalian taxa and may present simultaneously within a single individual. Macroscopic pathological changes are most commonly observed in the liver, spleen, gastrointestinal system, and mesenteric lymph nodes.

#### 5.1.2. Gross Pathology of Birds

In birds, multifocal white-yellow nodules have been reported in the liver (65.5%; 19/29), spleen (51.7%; 15/29), kidneys (13.8%; 4/29), pericardium (6.9%; 2/29), lungs (6.9%; 2/29), and air sacs (6.9%; 2/29) (see Appendix B, Table A4).

The most common lesions of the respiratory system are white-yellow nodules on the lungs and/or air sacs and pulmonary edema (see Appendix B, Table A4). In birds, the involvement of the air sacs has been reported, with lesions including orange spotting, serous effusion, or miliary nodules [29]. 

Similar to what has been observed in mammals, a combination of multifocal necrotizing splenitis and hepatitis appears to be common [6,7,18]. The spleen may be enlarged and contain white-yellow nodules, but hyperemia has not been reported in birds (see Appendix B, Table A4). Similar to the spleen, the liver is also frequently affected and may be enlarged and covered in white-yellow nodules. Unlike mammals, hepatic hyperemia has not been reported (see Appendix B, Table A4).

Inflammation in parts of the gastrointestinal tract is reported frequently, but the diagnosis is rarely confirmed histologically. Gastrointestinal tract wall edema has been reported in two Fischer’s turacos [29]. Moreover, enlargement of the cecal tonsils has been reported in zebra finches (*Poephila guttata*) and canaries (*Serinus canaria*) [42].

Renal lesions in birds may be present. White-yellow nodules are reported in a yellow-headed amazon, a blue-fronted amazon, a keel-billed toucan (*Ramphastos sulfuratus*), and a Von der Decken’s hornbill (*Tockus deckeni*) (see Appendix B, Table A4).

Reported cardiac lesions differ from those observed in mammals. No hemorrhages are described. Instead, systemic venous congestion has been reported in chestnut-mandibled (*Ramphastos swainsonii*) and keel-billed toucans [7], and the presence of white-yellow nodules has been reported in yellow-headed and blue-fronted amazons [5]. Coelomic effusion has only been reported in a single Fischer’s turaco (*Tauraco fischeri*) [29].

As can be seen in mammals, macroscopic pathological changes in birds most frequently occur in the liver, spleen, and gastrointestinal system without an appreciable distinction between types 1 and 2 lesions. Larger datasets may allow for the identification of more subtle taxonomical predispositions in the future.

### 5.2. Histopathology

In one study, 94.4% (17/18) of cases demonstrated varying levels of hemosiderosis. These cases occurred in silvery marmosets (*n* = 4), a white-faced saki (*n* = 1), scimitar-horned oryx (*n* = 4), impalas (*Aepyceros melampus*) (*n* = 2), toco toucans (*Ramphastos toco*) (*n* = 1), Fischer’s turacos (*n* = 2), Von der Decken’s hornbills (*n* = 1), tarictic hornbills (*Penelopides panini*) (*n* = 2), and Sulawesi hornbills *(Rhabdotorrhinus exarhatus)* (*n* = 1). Only one animal, a scimitar-horned oryx, had no observed hemosiderosis [29]. A correlation between the amount of stainable iron and the size of bacterial lesions in the liver of parrots that died from systemic pseudotuberculosis has been observed but only in animals with type 2 lesions [42].

#### 5.2.1. Histopathology of Mammals

The white-yellow nodules observed in the liver and spleen of a paca were described as multifocal necrosis. These lesions contained a central bacterial colony surrounded by necrotic cellular debris, which was surrounded by vacuolated macrophages and neutrophils. Along the perimeter of the lesions, hypereosinophilic necrotic hepatocytes or splenic parenchyma was observed [67]. In one study, these multifocal areas of coagulative and lytic hepatic necrosis with neutrophilic inflammation were reported in different taxonomic groups in 94.4% (*n* = 17/18) of cases, including avian species [29]. 

Micro-abscesses have been observed in the lamina propria of the jejunum and ileum and have been found inside Peyer’s patches in cervids [8]. In squirrel monkeys, severe congestion and focal necrosis with the infiltration of neutrophils, macrophages, and lymphocytes were observed in the small intestine, colon, and cecum. In the small and large intestines, lesions near the Peyer’s patches and solitary lymphatic nodules were severe and were accompanied by ulceration of the mucosa and hemorrhage [66]. Histopathology of the digestive tract of a paca revealed similar closely packed bacterial colonies in areas of mucosal necrosis. In these areas, the mucosa was diffusely collapsed and accumulations of fibrin, neutrophils, macrophages, and few lymphocytes were present, as well as hemorrhage and edema. The foci were often associated with lymphoid tissue, which also demonstrated necrosis [67]. Lesions in the gastrointestinal tract may extend into the submucosa [10,67]. Additionally, desquamation of the mucosal epithelium, congestion, hemorrhage, and numerous bacterial colonies may be observed in the gastrointestinal wall [10]. 

As described above, the nodules of focal necrosis may contain large clusters of Gram-negative bacilli [7]. Whereas *Y. pseudotuberculosis* bacteria are typically rod-shaped, aberrant morphologies have been described in squirrel monkeys and an African lion. These morphologies may present as globular bodies of 4–5 μm diameter with a fine granular structure or basophilic filaments. These morphologies may be present alongside rod-shaped bacteria in the same lesions and may be misdiagnosed as fungi or protozoa. As a result, special staining techniques, such as immunohistochemistry specific to O:6, may be required to confirm the diagnosis [45,66]. It has been hypothesized that these morphological changes were induced by β-lactam antibiotic treatment, which may have altered the structural integrity of the cell wall [45,66].

Myocardial degeneration and pulmonary edema were reported in a squirrel monkey [10]. 

Mesenteric lymph nodes are frequently affected, may be edematous, and may contain a large number of neutrophils and macrophages mixed with bacterial colonies [10].

#### 5.2.2. Histopathology of Birds

In birds, histological examination of the white-yellow nodules observed in various organs demonstrates that they are composed of acute severe coagulative necrosis with the infiltration of heterophils mixed with fibrin and dense colonies of bacteria [43]. In Amazon parrots, yellow nodules on the liver, kidney cortex, lungs, spleen, and pericardium were identified as microgranulomas, similarly surrounded by a strong heterophilic reaction [5]. Inflammatory foci may vary in size, with one study reporting a pronounced cellular response in only 30% of lesions in different bird species [42]. This study subdivided lesions into three types: (1) small lesions (<100 mm) containing a large number of bacteria and little cellular response, (2) intermediate lesions (100–200 mm) containing a central amorphous eosinophilic area of necrosis surrounded by heterophils, and (3) large granulomas (>200 mm) with a central necrotic zone surrounded by giant cells [42].

Enteritis is not unique to mammals as lymphocytic enteritis and congestion of the lamina propria have been reported in Eurasian collared doves (*Streptopelia decaocto*) [43]. Moreover, in Eurasian collared doves, lung changes were characterized by mild edema and diffuse distribution of dense bacterial colonies with little inflammatory response [43]. 

## 6. Diagnostics

Ante-mortem diagnosis may be challenging as clinical signs are generally non-specific, if noticeably present at all. The presence of *Y. pseudotuberculosis* may be demonstrated by means of PCR or bacterial culture. Regardless of the diagnostic method, it should be considered that *Y. pseudotuberculosis* may be present in the feces of healthy animals.

PCR assays for *Y. pseudotuberculosis* may be used to rapidly demonstrate the presence of the bacterium in a sample and may allow for the detection of the pYV virulence plasmid [29,68]. Moreover, a SYBR Green and TaqMan multiplex real-time PCR has been developed for the simultaneous detection and differentiation of highly pathogenic *Y. enterocolitica*, low pathogenic *Y. enterocolitica*, and *Y. pseudotuberculosis*, which may yield results within two to three hours [69]. PCR tests are commonly used on bacterial cultures or post-mortem as *Y. pseudotuberculosis* may also be present in the feces of healthy individuals. However, these PCR tests may not be readily available to the veterinary clinician and can be cost-prohibitive. 

*Y. pseudotuberculosis* may be isolated from various tissues on necropsy, such as the liver, spleen, lungs, and kidneys [16]. It is recommended to sample multiple organs as the bacteria may not be consistently isolated from a single organ and may be isolated from organs without macroscopic changes [7]. 

Isolation from feces may be difficult as growth is often suppressed through competitive inhibition by other gastrointestinal bacteria with routine bacterial isolation methods [29]. Moreover, selective media favoring *Y. pseudotuberculosis* growth are not available. Cold enrichment (4–10 °C) may improve the growth of *Y. pseudotuberculosis*; however, this may take up to 10–21 days, limiting its applicability in clinical settings [68]. More importantly, fecal samples do not always reveal shedding of *Y. pseudotuberculosis*, making it difficult to monitor the infection status by this means [6]. Nevertheless, despite the likely underestimation of the true prevalence, fecal analysis may constitute a non-invasive method to monitor carrier status in asymptomatic animals [70].

It can be concluded that PCR techniques provide a reliable method for detecting *Y. pseudotuberculosis* that is faster than bacterial culture. Although bacterial culture remains a viable alternative when PCR is cost-prohibitive or unavailable, the faster results provided by PCR may prove crucial in acute cases of yersiniosis where timely diagnosis is critical. For monitoring purposes, however, bacterial culture may be adequate.

## 7. Treatment and Prophylaxis

### 7.1. Treatment

Yersiniosis is often characterized as an acute condition with a rapid onset. Most animals are either found dead or require euthanasia within 24 h of the onset of clinical signs [6,29,32]. Moreover, multidrug resistance encoded by conjugative plasmids has been described for *Y. pseudotuberculosis* [71]. However, even in cases where antibiotic treatment is based on antimicrobial susceptibility testing, animals still frequently succumb to the disease [6,48]. Nevertheless, fluoroquinolones appear to be most effective in reducing mortality. Treatment with oral enrofloxacin conferred full protection against clinical disease in experimentally infected canaries, and IV marbofloxacin has been successfully used in combination with potentiated amoxicillin to treat yersiniosis in a domestic cat (*Felis catus*) [72,73]. The metaphylactic use of antibiotics during an outbreak has been used, but its efficacy in preventing infection has not been demonstrated [32].

Prompt initiation of antibiotic treatment is essential in managing acute yersiniosis, and veterinarians should not delay treatment while awaiting antimicrobial susceptibility test results. Fluoroquinolones are considered the most effective antibiotics, although their use may be subject to local regulations. Despite the limited discussion of supportive care in the peer-reviewed literature, it remains a critical component of the treatment plan and should be tailored to the individual case. 

In light of emerging microbial resistance, there is a critical need for novel therapeutic strategies. Treatment with strictly lytic *Y. pseudotuberculosis*-specific bacteriophages has been proposed as these bacteriophages are readily isolated from various zoological species, particularly birds, and are effective against multidrug-resistant bacteria. This treatment, however, is still in the experimental phase and is not yet accessible to veterinary professionals [74]. 

Future research should investigate the efficacy of metaphylactic antibiotic use during outbreaks. Additionally, trials assessing the effectiveness of *Y. pseudotuberculosis*-specific bacteriophages are warranted, particularly given the emergence of multidrug resistance in *Y. pseudotuberculosis* and the increasing importance of antimicrobial stewardship.

### 7.2. Vaccination

Yersiniavax^®^ (MSD Animal Health, Upper Hutt, New Zealand) is an inactivated vaccine containing serotypes 1 to 3. It is registered for the immunization of deer, and two 2 mL doses should be administered subcutaneously in the neck 3–6 weeks apart. This vaccine induces a robust antibody response in red deer (*Cervus elaphus*), although maternal immunity has been demonstrated to interfere with successful immunization in deer younger than 12 weeks [75]. Retrospective analysis has demonstrated that Yersiniavax^®^ successfully reduces the incidence rate of clinical yersiniosis by at least 66% in this species [76]. No information exists on the off-label use of this vaccine in other species.

A killed whole-cell vaccine composed of serotype 1 to 6 isolates, Pseudovac^®^ (Faculty of Veterinary Medicine, Utrecht University, The Netherlands), is available for subcutaneous immunization. This vaccine is updated annually and contains twenty different *Y. pseudotuberculosis* strains isolated from outbreaks in different animal species and regions. Despite anecdotal evidence of outbreak control and in spite of being able to induce a strong humoral immune response, its efficacy is not well evaluated [48,77]. Pseudovac^®^ failed to confer any protection in guinea pigs (*Cavia porcellus*) challenged with a high inoculum (10^9^ CFU) of the highly virulent IP32953 strain in 2010 [77]. 

In the past, a formol-killed vaccine adsorbed onto aluminum hydroxide gel has been administered to colonies of silvery marmosets, white-headed marmosets (*Callithrix geoffroyi*), and Goeldi’s marmosets (*Callimico goeldii*). The vaccine contained a titer of approximately 1.04 × 10^11^ CFU/mL and failed to provide significant protection when administered IM twice with a 30-day interval followed by an annual booster vaccination [34].

Alternatively, single or double oral inoculation of the avirulent IP32680 strain in guinea pigs has been demonstrated to induce 50% and 83% protection, respectively, against a challenge with a high inoculum (10^9^ CFU) of the highly virulent IP32953 *Y. pseudotuberculosis* strain. In this challenge, it was observed that immunoglobulins produced against IP32680 also recognized strains of other serotypes, suggesting that cross-immunity may be achieved [77]. 

Live vaccines may stimulate the immune system for a prolonged period of time as antigens are produced de novo as long as the bacteria persist in the host. Moreover, the antigens expressed in vivo may differ from those expressed in vitro. The observed efficacy of live vaccines may also be explained by the stimulation of both cellular and humoral immunity, whereas killed vaccines primarily stimulate humoral immunity [77]. In mice (*Mus musculus*) challenged with 10^7^ cells of the RIMD2503048 serotype O:4b strain, double vaccination at a one-week interval with 100 mg recombinant YadA conferred 100% protection [18]. As stated before, YadA has various functions in the pathogenesis, such as enabling *Y. pseudotuberculosis* to adhere to target cells and inhibiting phagocytosis by host cells. As a result, YadA-specific immunoglobulins may reduce hematogenous spread and the risk of sepsis [18].

Identifying risk factors associated with yersiniosis is crucial due to the challenges associated with the treatment of yersiniosis, as well as the unknown efficacy of the available Pseudovac^®^ vaccine. Although Yersiniavax^®^ appears to be moderately effective, no information exists on its efficacy and safety profile in species other than red deer. Nevertheless, an emergency booster vaccination in case of an epizootic event may be considered an alternative to metaphylactic antibiotic treatment for animals without clinical signs of disease. Live attenuated vaccines may constitute effective alternatives but are not currently licensed. 

Practical obstacles to the use of these vaccines may include limited regional availability and local legislation and regulations. Additionally, the wide range of animal species susceptible to yersiniosis could pose a financial barrier for institutions aiming to prophylactically immunize all at-risk species. Therefore, the decision to vaccinate against *Y. pseudotuberculosis* should always be based on a thorough risk assessment tailored to the zoological institution. Furthermore, prophylactic vaccination should complement, rather than replace, husbandry practices designed to minimize risk factors associated with yersiniosis, such as effective pest control and stress reduction. 

Given the widespread occurrence and high mortality rates associated with yersiniosis, along with the challenges in its treatment, the development of an effective vaccine is paramount to safeguarding the health of captive non-domestic animals globally. Future research should prioritize identifying suitable strains for the development of these vaccines and subsequently establishing their efficacy and safety profiles.

## 8. Conclusions

This review summarizes the current state of knowledge on *Y. pseudotuberculosis* infections across different taxa in zoological settings. The agent and its virulence factors are discussed, as well as routes of transmission, risk factors for clinical yersiniosis, symptoms, port-mortem observations, diagnostic options, treatment options, and prophylaxis. 

Yersiniosis remains a recurring disease in zoological institutions worldwide. Due to its often rapid progression, future research should focus on developing methods for early and rapid detection and diagnosis, as well as more effective treatment and prophylactic strategies. Additionally, the roles of various vector species and asymptomatic carrier animals require further investigation. Identifying additional risk factors will aid in creating more effective biosecurity and prophylactic plans to reduce the incidence of yersiniosis.

## Data Availability

Data are available upon reasonable request.

## Appendix A

**Table A1:** An overview of clinical signs as reported in the referred literature associated with *Y. pseudotuberculosis* in mammalian cases in zoological institutions.

Order	Family	Species	Number of Animals (*n*)	Sex (M/F/NA)	Age (Years) Avg. (Range)	Symptoms (Y/N/NA)	Anorexia/Hyporexia (Y/N/NA or Asymptomatic)	Apathy/Lethargy (Y/N/NA or Asymptomatic)	Diarrhea (Y/N/NA or Asymptomatic)	Ataxia (Y/N/NA or Asymptomatic)	Paralysis/Paresis (Y/N/NA or Asymptomatic)	**Abdominal Distension** **(Y/N/NA or Asymptomatic)**	**Jaundice** **(Y/N/NA or Asymptomatic)**	**Dyspnea** **(Y/N/NA or Asymptomatic)**	**Death** **(Yes/No/NA)**	**Reference**
Artiodactyla	Bovidae	**Gemsbok** **(*Oryx gazella*)**	1	0/1/0	NA	**100.0%** **(1/0/0)**	0.0% (0/1/0)	**100.0%** **(1/0/0)**	0.0% (0/1/0)	**100.0%** **(1/0/0)**	0.0% (0/1/0)	0.0% (0/1/0)	0.0% (0/1/0)	0.0% (0/1/0)	**100.0%** **(1/0/0)**	[29]
		**Impala** **(*Aepyceros melampus*)**	2	2/0/0	NA	**100.0%** **(2/0/0)**	0.0% (0/2/0)	**50.0%** **(1/1/0)**	0.0% (0/2/0)	0.0% (0/2/0)	0.0% (0/2/0)	0.0% (0/2/0)	0.0% (0/2/0)	0.0% (0/2/0)	**100.0%** **(2/0/0)**	[29]
		**Scimitar-horned oryx** **(*Oryx dammah*)**	4	3/1/0	NA	**75.0%** **(3/1/0)**	**33.3%** **(1/2/1)**	**100.0%** **(3/0/1)**	**33.3%** **(1/2/1)**	**33.3%** **(1/2/1)**	0.0% (0/3/1)	0.0% (0/3/1)	0.0% (0/3/1)	0.0% (0/3/1)	**100.0%** **(4/0/0)**	[29]
	Cervidae	**Axis deer** **(*Axis axis*)**	1	0/1/0	NA	0.0% (0/1/0)	NA (0/0/1)	NA (0/0/1)	NA (0/0/1)	NA (0/0/1)	NA (0/0/1)	NA (0/0/1)	NA (0/0/1)	NA (0/0/1)	**100.0%** **(1/0/0)**	[29]
		**Chinese water deer** **(*Hydropotes inermis*)**	1	0/1/0	NA	0.0% (0/1/0)	NA (0/0/1)	NA (0/0/1)	NA (0/0/1)	NA (0/0/1)	NA (0/0/1)	NA (0/0/1)	NA (0/0/1)	NA (0/0/1)	**100.0%** **(1/0/0)**	[29]
		**Fallow deer** **(*Dama dama*)**	5	1/2/2	0.33 (0.33)	**100.0%** **(1/0/4)**	0.0% (0/1/4)	0.0% (0/1/4)	**100.0%** **(1/0/4)**	0.0% (0/1/4)	0.0% (0/1/4)	0.0% (0/1/4)	0.0% (0/1/4)	0.0% (0/1/4)	**100.0%** **(5/0/0)**	[8,29]
		**Elk** **(*Cervus canadiensis*)**	6	2/0/4	0.42 (0.42)	NA (0/0/6)	NA (0/0/6)	NA (0/0/6)	NA (0/0/6)	NA (0/0/6)	NA (0/0/6)	NA (0/0/6)	NA (0/0/6)	NA (0/0/6)	**100.0%** **(6/0/0)**	[8]
		**Red deer** **(*Cervus elaphus*)**	13	0/3/10	0.49 (0.33–1.67)	NA (0/0/13)	NA (0/0/13)	NA (0/0/13)	NA (0/0/13)	NA (0/0/13)	NA (0/0/13)	NA (0/0/13)	NA (0/0/13)	NA (0/0/13)	**100.0%** **(13/0/0)**	[8]
		**Red deer × Elk** **(*Cervus elaphus* × *Cervus canadiensis*)**	2	1/0/1	0.42 (0.42)	NA (0/0/2)	NA (0/0/2)	NA (0/0/2)	NA (0/0/2)	NA (0/0/2)	NA (0/0/2)	NA (0/0/2)	NA (0/0/2)	NA (0/0/2)	**100.0%** **(2/0/0)**	[8]
		**Southern pudu** **(*Pudu puda*)**	1	0/0/1	NA	**100.0%** **(1/0/0)**	0.0% (0/1/0)	0.0% (0/1/0)	**100.0%** **(1/0/0)**	0.0% (0/1/0)	0.0% (0/1/0)	0.0% (0/1/0)	0.0% (0/1/0)	0.0% (0/1/0)	**100.0%** **(1/0/0)**	[74]
Carnivora	Felidae	**African lion** **(*Panthera leo*)**	3	2/1/0	16 (14–17)	**100.0%** **(3/0/0)**	**100.0%** **(3/0/0)**	**100.0%** **(3/0/0)**	0.0% (0/3/0)	0.0% (0/3/0)	0.0% (0/3/0)	0.0% (0/3/0)	0.0% (0/3/0)	**66.7%** **(2/1/0)**	**66.7%** **(2/1/0)**	[45]
		**Cougar** **(*Felis concolor*)**	1	1/0/0	14 (14)	**100.0%** **(1/0/0)**	**100.0%** **(1/0/0)**	0.0% (0/1/0)	0.0% (0/1/0)	0.0% (0/1/0)	0.0% (0/1/0)	0.0% (0/1/0)	**100.0%** **(1/0/0)**	0.0% (0/1/0)	**100.0%** **(1/0/0)**	[44]
Chiroptera	Phyllostomidae	**Seba’s short-tailed bat** **(*Carollia perspicillata*)**	2	1/1/0	NA	**50.0%** **(1/1/0)**	0.0% (0/1/1)	0.0% (0/1/1)	0.0% (0/1/1)	0.0% (0/1/1)	0.0% (0/1/1)	0.0% (0/1/1)	0.0% (0/1/1)	0.0% (0/1/1)	**100.0%** **(2/0/0)**	[16]
	Pteropodidae	**Egyptian rousette bat *** **(*Rousettus aegyptiacus*)**	92	17/13/62	NA	**7.6%** **(7/85/0)**	NA (0/0/92)	NA (0/0/92)	NA (0/0/92)	NA (0/0/92)	NA (0/0/92)	NA (0/0/92)	NA (0/0/92)	NA (0/0/92)	**100.0%** **(92/0/0)**	[31,47]
Hystricomorpha	Cuniculidae	**Paca** **(*Cuniculus paca*)**	1	0/1/0	2 (2)	0.0% (0/1/0)	NA (0/0/1)	NA (0/0/1)	NA (0/0/1)	NA (0/0/1)	NA (0/0/1)	NA (0/0/1)	NA (0/0/1)	NA (0/0/1)	**100.0%** **(1/0/0)**	[67]
Macroscelidea	Macroscelididae	**Short-eared elephant shrew** **(*Macroscelides proboscideus*)**	1	0/0/1	NA	**100.0%** **(1/0/0)**	0.0% (0/1/0)	**100.0%** **(1/0/0)**	0.0% (0/1/0)	0.0% (0/1/0)	0.0% (0/1/0)	0.0% (0/1/0)	0.0% (0/1/0)	0.0% (0/1/0)	**100.0%** **(1/0/0)**	[6]
Primates	Atelidae	**Brown-headed spider monkey** **(*Ateles fusciceps*)**	2	0/0/2	NA	**100.0%** **(2/0/0)**	**50.0%** **(1/1/0)**	**100.0%** **(2/0/0)**	0.0% (0/2/0)	0.0% (0/2/0)	0.0% (0/2/0)	0.0% (0/2/0)	0.0% (0/2/0)	0.0% (0/2/0)	**100.0%** **(2/0/0)**	[6]
	Cebidae	**Squirrel monkey** **(*Saimiri* sp.)**	12	0/0/12	NA	**25.0%** **(3/9/0)**	**100.0%** **(3/0/9)**	0.0% (0/3/9)	0.0% (0/3/9)	0.0% (0/3/9)	0.0% (0/3/9)	0.0% (0/3/9)	0.0% (0/3/9)	0.0% (0/3/9)	**100.0%** **(12/0/0)**	[7]
		**Bolivian squirrel monkey** **(*Saimiri boliviensis*)**	1	1/0/0	<1 (<1)	**100.0%** **(1/0/0)**	0.0% (0/1/0)	**100.0%** **(1/0/0)**	**100.0%** **(1/0/0)**	0.0% (0/1/0)	0.0% (0/1/0)	0.0% (0/1/0)	0.0% (0/1/0)	0.0% (0/1/0)	**100.0%** **(1/0/0)**	[10]
		**Silvery marmoset** **(*Mico argentatus*)**	5	2/2/1	NA	**100.0%** **(5/0/0)**	**20.0%** **(1/4/0)**	**80.0%** **(4/1/0)**	0.0% (0/5/0)	**60.0%** **(3/2/0)**	**20.0%** **(1/4/0)**	0.0% (0/5/0)	0.0% (0/5/0)	0.0% (0/5/0)	**100.0%** **(5/0/0)**	[29]
	Pitheciidae	**White-faced saki** **(*Pithecia pithecia*)**	1	0/1/0	NA	**100.0%** **(1/0/0)**	0.0% (0/1/0)	**100.0%** **(1/0/0)**	0.0% (0/1/0)	0.0% (0/1/0)	0.0% (0/1/0)	**100.0%** **(1/0/0)**	0.0% (0/1/0)	0.0% (0/1/0)	**100.0%** **(1/0/0)**	[29]
Rodentia	Caviidae	**Chacoan mara** **(*Dolichotis salinicola*)**	1	0/0/1	NA	0.0% (0/1/0)	NA (0/0/1)	NA (0/0/1)	NA (0/0/1)	NA (0/0/1)	NA (0/0/1)	NA (0/0/1)	NA (0/0/1)	NA (0/0/1)	**100.0%** **(1/0/0)**	[6]
		**Patagonian mara** **(*Dolichotis patagonum*)**	2	0/0/2	NA	**50.0%** **(1/1/0)**	0.0% (0/1/1)	**100.0%** **(1/0/1)**	0.0% (0/1/1)	**100.0%** **(1/0/1)**	**100.0%** **(1/0/1)**	0.0% (0/1/1)	0.0% (0/1/1)	0.0% (0/1/1)	**100.0%** **(2/0/0)**	[6]
	Hydrochaeridae	**Capybara** **(*Hydrochoerus hydrochaeris*)**	9	6/1/2	0.28 (0.25–0.33)	**62.5%** **(5/3/1)**	**100.0%** **(5/0/4)**	**100.0%** **(5/0/4)**	**20.0%** **(1/4/4)**	0.0% (0/5/4)	0.0% (0/5/4)	0.0% (0/5/4)	0.0% (0/5/4)	0.0% (0/5/4)	**100.0%** **(9/0/0)**	[32,65]
	Sciuridae	**Japanese squirrel** **(*Sciurus lis*)**	1	0/1/0	2 (2)	**100.0%** **(1/0/0)**	0.0% (0/1/0)	0.0% (0/1/0)	**100.0%** **(1/0/0)**	0.0% (0/1/0)	0.0% (0/1/0)	0.0% (0/1/0)	0.0% (0/1/0)	0.0% (0/1/0)	**100.0%** **(1/0/0)**	[7]
		**Total**	**170**	**39/30/101**		**27.8%** **40/104/26**	**45.5%** **15/18/137**	**66.7%** **22/11/137**	**18.2%** **6/27/137**	**18.2%** **6/27/137**	**6.1%** **2/31/137**	**3.0%** **1/32/137**	**3.0%** **1/32/137**	**6.1%** **2/31/137**	**99.4%** **169/1/0**	

Both percentages and absolute numbers of cases in which clinical signs are described are presented. Reports published before 1995 were excluded from the analysis. NA is used to indicate that the data are not available for this species. * In Egyptian rousette bats, the number of animals displaying specific symptoms is not described. As a result, these data are considered to be unavailable in the row describing the total column occurrence.

**Table A2:** An overview of clinical signs as reported in the referred literature associated with *Y. pseudotuberculosis* in avian cases in zoological institutions.

Order	Family	Species	Number of Animals (*n*)	Sex (M/F/NA)	Age (Years) Avg. (Range)	Symptoms (Y/N/NA)	Anorexia/Hyporexia (Y/N/NA or Asymptomatic)	Apathy/Lethargy (Y/N/NA or Asymptomatic)	Diarrhea (Y/N/NA or Asymptomatic)	Death (Yes/No/NA)	Reference
Bucerotiformes	Bucerotidae	**Sulawesi hornbill** **(*Rhabdotorrhinus exarhatus*)**	1	1/0/0	NA	**100.0%** **(1/0/0)**	0.0% (0/1/0)	**100.0%** **(1/0/0**)	0.0% (0/1/0)	**100.0%** **(1/0/0)**	[29]
		**Tarictic hornbill** **(*Penelopides panini*)**	3	3/0/0	NA	**66.7%** **(2/1/0)**	**50.0%** **(1/1/1)**	**100.0%** **(2/0/1)**	0.0% (0/2/1)	**100.0%** **(3/0/0)**	[29]
		**Von der Decken’s hornbill** **(*Tockus deckeni*)**	1	1/0/0	NA	0.0% (0/1/0)	NA (0/0/1)	NA (0/0/1)	NA (0/0/1)	**100.0%** **(1/0/0)**	[29]
Columbiformes	Columbidae	**Eurasian collared dove** **(*Streptopelia decaocto*)**	4	0/2/2	NA	0.0% (0/4/0)	NA (0/0/4)	NA (0/0/4)	NA (0/0/4)	**100.0%** **(4/0/0)**	[43]
		**New Zealand wood pigeon** **(*Hemiphaga novaeseelandiae*)**	3	0/0/3	NA	NA (0/0/3)	NA (0/0/3)	NA (0/0/3)	NA (0/0/3)	**100.0%** **(3/0/0)**	[42]
Musophagiformes	Musophagidae	**Fischer’s turaco** **(*Tauraco fischeri*)**	2	1/1/0	NA	0.0% (0/2/0)	NA (0/0/2)	NA (0/0/2)	NA (0/0/2)	**100.0%** **(2/0/0)**	[29]
Passeriformes	Estrildidae	**Zebra finch** **(*Poephila guttata*)**	2	0/0/2	NA	NA (0/0/2)	NA (0/0/2)	NA (0/0/2)	NA (0/0/2)	**100.0%** **(2/0/0)**	[42]
	Fringillidae	**Canary** **(*Serinus canaria*)**	6	0/0/6	NA	NA (0/0/6)	NA (0/0/6)	NA (0/0/6)	NA (0/0/6)	**100.0%** **(6/0/0)**	[42]
Piciformes	Ramphastidae	**Chestnut-mandibled toucan** **(*Ramphastos swainsonii*)**	1	0/1/0	NA	0.0% (0/1/0)	NA (0/0/1)	NA (0/0/1)	NA (0/0/1)	**100.0%** **(1/0/0)**	[7]
		**Keel-billed toucan** **(*Ramphastos sulfuratus*)**	2	1/1/0	NA	0.0% (0/2/0)	NA (0/0/2)	NA (0/0/2)	NA (0/0/2)	**100.0%** **(2/0/0)**	[7]
		**Toco toucan** **(*Ramphastos toco*)**	1	0/1/0	NA	0.0% (0/1/0)	NA (0/0/1)	NA (0/0/1)	NA (0/0/1)	**100.0%** **(1/0/0)**	[29]
Psittaciformes	Psittacidae	**Blue-fronted amazon** **(*Amazona aestiva*)**	1	0/1/0	7 (7)	**100.0%** **(1/0/0)**	0.0% (0/1/0)	**100.0%** **(1/0/0)**	**100.0%** **(1/0/0)**	**100.0%** **(1/0/0)**	[5]
		**Kaka** **(*Nestor meriondalis*)**	1	0/0/1	NA	NA (0/0/1)	NA (0/0/1)	NA (0/0/1)	NA (0/0/1)	**100.0%** **(1/0/0)**	[42]
		**Yellow-headed amazon** **(*Amazona oratrix*)**	1	0/1/0	0.67 (0.67)	**100.0%** **(1/0/0)**	0.0% (0/1/0)	**100.0%** **(1/0/0)**	**100.0%** **(1/0/0)**	**100.0%** **(1/0/0)**	[5]
	Psittaculidae	**Budgerigar** **(*Melopsittacus undulatus*)**	1	0/0/1	NA	NA (0/0/1)	NA (0/0/1)	NA (0/0/1)	NA (0/0/1)	**100.0%** **(1/0/0)**	[42]
		**Rainbow lorikeet** **(*Trichoglossus mollucanus*)**	1	0/0/1	NA	NA (0/0/1)	NA (0/0/1)	NA (0/0/1)	NA (0/0/1)	**100.0%** **(1/0/0)**	[42]
		**Total**	**31**	**7/8/16**		**29.4%** **(5/12/14)**	**20.0%** **(1/4/26)**	**100.0%** **(5/0/26)**	**40.0%** **(2/3/26)**	**100.0%** **(31/0/0)**	

Both percentages and absolute numbers of cases in which clinical signs are described are presented. Reports older than 1995 were excluded from the analysis. NA is used to indicate that the data are not available for this species.

## Appendix B

**Table A3:** An overview of gross post-mortem observations as reported in the referred literature in mammals that died from *Y. pseudotuberculosis* in zoological institutions.

								Respiratory System					Lymphoid Tissue					**Liver**		
Order	Family	Species	Number of Animals (*n*)	Sex (M/F/NA)	Age (Years) Avg. (Range)	Body Condition (Good/Poor/NA)	Pleural or Peritoneal Effusion (Y/N/NA)	White-Yellow Nodules on Lungs (Y/N/NA)	(Broncho)pneumonia (Y/N/NA)	Pulmonary Hyperemia (Y/N/NA)	Pulmonary Edema (Y/N/NA)	Pulmonary Petechiae (Y/N/NA)	White-Yellow Nodules on Mesenteric Lymph Nodes (Y/N/NA)	Lymphadenopathy of Mesenteric Lymph Nodes (Y/N/NA)	Splenomegaly (Y/N/NA)	White-Yellow Nodules on Spleen (Y/N/NA)	**Splenic Hyperemia** **(Y/N/NA)**	**Hepatic Hyperemia** **(Y/N/NA)**	**White or Yellow Nodules on Liver** **(Y/N/NA)**	**Reference**
Artiodactyla	Bovidae	**Gemsbok** **(*Oryx gazella*)**	1	0/1/0	NA	0/1/0	0.0% (0/1/0)	0.0% (0/1/0)	0.0% (0/1/0)	0.0% (0/1/0)	0.0% (0/1/0)	0.0% (0/1/0)	0.0% (0/1/0)	**100.0%** **(1/0/0)**	0.0% (0/1/0)	0.0% (0/1/0)	0.0% (0/1/0)	0.0% (0/1/0)	0.0% (0/1/0)	[29]
		**Impala** **(*Aepyceros melampus*)**	2	2/0/0	NA	0/2/0	0.0% (0/2/0)	0.0% (0/2/0)	0.0% (0/2/0)	0.0% (0/2/0)	0.0% (0/2/0)	0.0% (0/2/0)	0.0% (0/2/0)	**100.0%** **(2/0/0)**	0.0% (0/2/0)	0.0% (0/2/0)	0.0% (0/2/0)	0.0% (0/2/0)	0.0% (0/2/0)	[29]
		**Scimitar-horned oryx** **(*Oryx dammah*)**	4	3/1/0	NA	0/4/0	0.0% (0/4/0)	0.0% (0/4/0)	0.0% (0/4/0)	0.0% (0/4/0)	0.0% (0/4/0)	0.0% (0/4/0)	0.0% (0/4/0)	**100.0%** **(4/0/0)**	0.0% (0/4/0)	0.0% (0/4/0)	0.0% (0/4/0)	0.0% (0/4/0)	**50.0%** **(2/2/0)**	[29]
	Cervidae	**Axis deer** **(*Axis axis*)**	1	0/1/0	NA	0/1/0	0.0% (0/1/0)	0.0% (0/1/0)	0.0% (0/1/0)	0.0% (0/1/0)	0.0% (0/1/0)	0.0% (0/1/0)	0.0% (0/1/0)	0.0% (0/1/0)	0.0% (0/1/0)	0.0% (0/1/0)	0.0% (0/1/0)	0.0% (0/1/0)	0.0% (0/1/0)	[29]
		**Chinese water deer** **(*Hydropotes inermis*)**	1	0/1/0	NA	0/1/0	0.0% (0/1/0)	0.0% (0/1/0)	0.0% (0/1/0)	0.0% (0/1/0)	0.0% (0/1/0)	0.0% (0/1/0)	0.0% (0/1/0)	0.0% (0/1/0)	0.0% (0/1/0)	0.0% (0/1/0)	0.0% (0/1/0)	0.0% (0/1/0)	0.0% (0/1/0)	[29]
		**Fallow deer** **(*Dama dama*)**	5	1/2/2	0.33 (0.33)	0/1/4	0.0% (0/1/4)	0.0% (0/1/4)	0.0% (0/1/4)	0.0% (0/1/4)	0.0% (0/1/4)	0.0% (0/1/4)	0.0% (0/1/4)	**100.0%** **(1/0/4)**	0.0% (0/1/4)	0.0% (0/1/4)	0.0% (0/1/4)	0.0% (0/1/4)	0.0% (0/1/4)	[8,29]
		**Elk** **(*Cervus canadiensis*)**	6	2/0/4	0.42 (0.42)	0/0/6	NA (0/0/6)	NA (0/0/6)	NA (0/0/6)	NA (0/0/6)	NA (0/0/6)	NA (0/0/6)	NA (0/0/6)	NA (0/0/6)	NA (0/0/6)	NA (0/0/6)	NA (0/0/6)	NA (0/0/6)	NA (0/0/6)	[8]
		**Red deer** **(*Cervus elaphus*)**	13	0/3/10	0.49 (0.33–1.67)	0/0/13	NA (0/0/13)	NA (0/0/13)	NA (0/0/13)	NA (0/0/13)	NA (0/0/13)	NA (0/0/13)	NA (0/0/13)	NA (0/0/13)	NA (0/0/13)	NA (0/0/13)	NA (0/0/13)	NA (0/0/13)	NA (0/0/13)	[8]
		**Red deer × Elk** **(*Cervus elaphus* × *Cervus canadiensis*)**	2	1/0/1	0.42 (0.42)	0/0/2	NA (0/0/2)	NA (0/0/2)	NA (0/0/2)	NA (0/0/2)	NA (0/0/2)	NA (0/0/2)	NA (0/0/2)	NA (0/0/2)	NA (0/0/2)	NA (0/0/2)	NA (0/0/2)	NA (0/0/2)	NA (0/0/2)	[8]
		**Southern pudu** **(*Pudu puda*)**	1	0/0/1	NA	0/0/1	0.0% (0/1/0)	0.0% (0/1/0)	0.0% (0/1/0)	0.0% (0/1/0)	0.0% (0/1/0)	0.0% (0/1/0)	0.0% (0/1/0)	0.0% (0/1/0)	0.0% (0/1/0)	0.0% (0/1/0)	0.0% (0/1/0)	0.0% (0/1/0)	0.0% (0/1/0)	[74]
Carnivora	Felidae	**African lion** **(*Panthera leo*)**	3	2/1/0	16 (14–17)	1/1/1	**50.0%** **(1/1/1)**	0.0% (0/2/1)	**50.0%** **(1/1/1)**	**100.0%** **(2/0/1)**	**100.0%** **(2/0/1)**	**100.0%** **(2/0/1)**	**100.0%** **(2/0/1)**	0.0% (0/2/1)	0.0% (0/2/1)	0.0% (0/2/1)	0.0% (0/2/1)	0.0% (0/2/1)	**100.0%** **(2/0/1)**	[45]
		**Cougar** **(*Felis concolor*)**	1	1/0/0	14 (14)	0/0/1	0.0% (0/1/0)	0.0% (0/1/0)	0.0% (0/1/0)	0.0% (0/1/0)	0.0% (0/1/0)	0.0% (0/1/0)	0.0% (0/1/0)	0.0% (0/1/0)	0.0% (0/1/0)	0.0% (0/1/0)	0.0% (0/1/0)	0.0% (0/1/0)	**100.0%** **(1/0/0)**	[44]
Chiroptera	Phyllostomidae	**Seba’s short-tailed bat** **(*Carollia perspicillata*)**	2	1/1/0	NA	0/0/2	0.0% (0/2/0)	0.0% (0/2/0)	**100.0%** **(2/0/0)**	**100.0%** **(2/0/0)**	0.0% (0/2/0)	0.0% (0/2/0)	0.0% (0/2/0)	**50.0%** **(1/1/0)**	0.0% (0/2/0)	0.0% (0/2/0)	0.0% (0/2/0)	0.0% (0/2/0)	**100.0%** **(2/0/0)**	[16]
	Pteropodidae	**Egyptian rousette bat *** **(*Rousettus aegyptiacus*)**	92	17/13/62	NA	0/0/92	0.0% (0/42/50)	**33.3%** **(14/28/50)**	0.0% (0/42/50)	0.0% (0/42/50)	0.0% (0/42/50)	0.0% (0/42/50)	0.0% (0/42/50)	**52.4%** **(22/20/50)**	**28.6%** **(12/30/50)**	**28.6%** **(12/30/50)**	0.0% (0/42/50)	0.0% (0/42/50)	**40.5%** **(17/25/50)**	[31,47]
Hystricomorpha	Cuniculidae	**Paca** **(*Cuniculus paca*)**	1	0/1/0	2 (2)	1/0/0	0.0% (0/1/0)	0.0% (0/1/0)	**100.0%** **(1/0/0)**	**100.0%** **(1/0/0)**	0.0% (0/1/0)	0.0% (0/1/0)	0.0% (0/1/0)	**100.0%** **(1/0/0)**	0.0% (0/1/0)	**100.0%** **(1/0/0)**	0.0% (0/1/0)	0.0% (0/1/0)	**100.0%** **(1/0/0)**	[67]
Macroscelidea	Macroscelididae	**Short-eared elephant shrew** **(*Macroscelides proboscideus*)**	1	0/0/1	NA	0/0/1	0.0% (0/1/0)	0.0% (0/1/0)	0.0% (0/1/0)	0.0% (0/1/0)	0.0% (0/1/0)	0.0% (0/1/0)	0.0% (0/1/0)	0.0% (0/1/0)	0.0% (0/1/0)	0.0% (0/1/0)	0.0% (0/1/0)	0.0% (0/1/0)	**100.0%** **(1/0/0)**	[6]
Primates	Atelidae	**Brown-headed spider monkey** **(*Ateles fusciceps*)**	2	0/0/2	NA	0/0/2	0.0% (0/2/0)	0.0% (0/2/0)	0.0% (0/2/0)	0.0% (0/2/0)	0.0% (0/2/0)	0.0% (0/2/0)	0.0% (0/2/0)	**50.0%** **(1/1/0)**	**50.0%** **(1/1/0)**	**50.0%** **(1/1/0)**	0.0% (0/2/0)	0.0% (0/2/0)	**50.0%** **(1/1/0)**	[6]
	Cebidae	**Squirrel monkey** **(*Saimiri* sp.)**	13	0/0/12	NA	0/0/12	**100.0%** **(8/0/4)**	0.0% (0/8/4)	0.0% (0/8/4)	0.0% (0/8/4)	**100.0%** **(8/0/4)**	**100.0%** **(8/0/4)**	**100.0%** **(8/0/4)**	**100.0%** **(8/0/4)**	**100.0%** **(8/0/4)**	**100.0%** **(8/0/4)**	0.0% (0/8/4)	0.0% (0/8/4)	**100.0%** **(8/0/4)**	[7]
		**Bolivian squirrel monkey** **(*Saimiri boliviensis*)**	1	1/0/0	<1 (<1)	0/0/1	**100.0%** **(1/0/0)**	0.0% (0/1/0)	0.0% (0/1/0)	0.0% (0/1/0)	0.0% (0/1/0)	0.0% (0/1/0)	0.0% (0/1/0)	**100.0%** **(1/0/0)**	**100.0%** **(1/0/0)**	**100.0%** **(1/0/0)**	0.0% (0/1/0)	0.0% (0/1/0)	**100.0%** **(1/0/0)**	[10]
		**Silvery marmoset** **(*Mico argentatus*)**	5	2/2/1	NA	0/5/0	0.0% (0/5/0)	**20.0%** **(1/4/0)**	0.0% (0/5/0)	0.0% (0/5/0)	0.0% (0/5/0)	0.0% (0/5/0)	0.0% (0/5/0)	**80.0%** **(4/1/0)**	0.0% (0/5/0)	0.0% (0/5/0)	0.0% (0/5/0)	0.0% (0/5/0)	**60.0%** **(3/2/0)**	[29]
	Pitheciidae	**White-faced saki** **(*Pithecia pithecia*)**	1	0/1/0	NA	0/1/0	0.0% (0/1/0)	0.0% (0/1/0)	0.0% (0/1/0)	0.0% (0/1/0)	0.0% (0/1/0)	0.0% (0/1/0)	0.0% (0/1/0)	0.0% (0/1/0)	0.0% (0/1/0)	0.0% (0/1/0)	0.0% (0/1/0)	0.0% (0/1/0)	**100.0%** **(1/0/0)**	[29]
Rodentia	Caviidae	**Chacoan mara** **(*Dolichotis salinicola*)**	1	0/0/1	NA	0/0/1	0.0% (0/1/0)	0.0% (0/1/0)	0.0% (0/1/0)	0.0% (0/1/0)	0.0% (0/1/0)	0.0% (0/1/0)	0.0% (0/1/0)	0.0% (0/1/0)	0.0% (0/1/0)	**100.0%** **(1/0/0)**	0.0% (0/1/0)	**100.0%** **(1/0/0)**	0.0% (0/1/0)	[6]
		**Patagonian mara** **(*Dolichotis patagonum*)**	2	0/0/2	NA	0/0/2	0.0% (0/2/0)	0.0% (0/2/0)	0.0% (0/2/0)	0.0% (0/2/0)	0.0% (0/2/0)	0.0% (0/2/0)	0.0% (0/2/0)	0.0% (0/2/0)	0.0% (0/2/0)	**50.0%** **(1/1/0)**	0.0% (0/2/0)	0.0% (0/2/0)	**50.0%** **(1/1/0)**	[6]
	Hydrochaeridae	**Capybara** **(*Hydrochoerus hydrochaeris*)**	9	6/1/2	0.28 (0.25–0.33)	9/0/0	**100.0%** **(5/0/4)**	**40.0%** **(2/3/4)**	**40.0%** **(2/3/4)**	**60.0%** **(3/2/4)**	0.0% (0/5/4)	0.0% (0/5/4)	**40.0%** **(2/3/4)**	**60.0%** **(3/2/4)**	**40.0%** **(2/3/4)**	0.0% (0/5/4)	**60.0%** **(3/2/4)**	**60.0%** **(3/2/4)**	0.0% (0/5/4)	[32,65]
	Sciuridae	**Japanese squirrel** **(*Sciurus lis*)**	1	0/1/0	2 (2)	0/1/0	0.0% (0/1/0)	0.0% (0/1/0)	0.0% (0/1/0)	0.0% (0/1/0)	0.0% (0/1/0)	0.0% (0/1/0)	0.0% (0/1/0)	0.0% (0/1/0)	**100.0%** **(1/0/0)**	**100.0%** **(1/0/0)**	0.0% (0/1/0)	0.0% (0/1/0)	**100.0%** **(1/0/0)**	[7]
		**Total**	**170**	**39/30/101**		**11/18/141**	**34.1%** **(15/29/126)**	**4.5%** **(2/42/126)**	**13.6%** **(6/38/126)**	**18.2%** **(8/36/126)**	**22.7%** **(10/34/126)**	**22.7%** **(10/34/126)**	**22.7%** **(10/34/126)**	**63.6%** **(28/16/126)**	**29.5%** **(13/31/126)**	**31.8%** **(14/30/126)**	**6.8%** **(3/41/126)**	**9.1%** **(4/40/126)**	**56.8%** **(25/19/126)**	
						**Liver**	**Gastrointestinal System**							**Kidneys**			**Adrenal Glands**	**Skin**	**Circulatory System**	
**Order**	**Family**	**Species**	**Number of Animals** **(*n*)**	**Sex** **(M/F/NA)**	**Age (Years) Avg.** **(Range)**	**Hepatomegaly** **(Y/N/NA)**	**White-Yellow Foci on Intestines** **(Y/N/NA)**	**Gastritis, Enteritis, Colitis, or Combination** **(Y/N/NA)**	**GI tract Hemorrhage** **(Y/N/NA)**		**GI Tract Wall Edema** **(Y/N/NA)**	**Stomatitis** **(Y/N/NA)**	**Glossitis** **(Y/N/NA)**	**Renal Hyperemia** **(Y/N/NA)**	**Renal Petechiae**	**White-Yellow Nodules on Kidneys** **(Y/N/NA)**	**Adrenalitis** **(Y/N/NA)**	**Petechial Hemorrhages** **(Y/N/NA)**	**Cardiac Petechiae** **(Y/N/NA)**	**Reference**
Artiodactyla	Bovidae	**Gemsbok** **(*Oryx gazella*)**	1	0/1/0	NA	0.0% (0/1/0)	0.0% (0/1/0)	**100.0%** **(1/0/0)**	0.0% (0/1/0)		0.0% (0/1/0)	0.0% (0/1/0)	0.0% (0/1/0)	0.0% (0/1/0)	0.0% (0/1/0)	0.0% (0/1/0)	0.0% (0/1/0)	0.0% (0/1/0)	0.0% (0/1/0)	[29]
		**Impala** **(*Aepyceros melampus*)**	2	2/0/0	NA	0.0% (0/2/0)	0.0% (0/2/0)	0.0% (0/2/0)	0.0% (0/2/0)		0.0% (0/2/0)	0.0% (0/2/0)	0.0% (0/2/0)	0.0% (0/2/0)	0.0% (0/2/0)	0.0% (0/2/0)	0.0% (0/2/0)	0.0% (0/2/0)	0.0% (0/2/0)	[29]
		**Scimitar-horned oryx** **(*Oryx dammah*)**	4	3/1/0	NA	0.0% (0/4/0)	0.0% (0/4/0)	**100.0%** **(4/0/0)**	0.0% (0/4/0)		0.0% (0/4/0)	0.0% (0/4/0)	0.0% (0/4/0)	0.0% (0/4/0)	0.0% (0/4/0)	0.0% (0/4/0)	0.0% (0/4/0)	0.0% (0/4/0)	0.0% (0/4/0)	[29]
	Cervidae	**Axis deer** **(*Axis axis*)**	1	0/1/0	NA	0.0% (0/1/0)	0.0% (0/1/0)	**100.0%** **(1/0/0)**	**100.0%** **(1/0/0)**		0.0% (0/1/0)	0.0% (0/1/0)	0.0% (0/1/0)	0.0% (0/1/0)	0.0% (0/1/0)	0.0% (0/1/0)	0.0% (0/1/0)	0.0% (0/1/0)	0.0% (0/1/0)	[29]
		**Chinese water deer** **(*Hydropotes inermis*)**	1	0/1/0	NA	**100.0%** **(1/0/0)**	0.0% (0/1/0)	0.0% (0/1/0)	0.0% (0/1/0)		0.0% (0/1/0)	0.0% (0/1/0)	0.0% (0/1/0)	0.0% (0/1/0)	0.0% (0/1/0)	0.0% (0/1/0)	0.0% (0/1/0)	0.0% (0/1/0)	0.0% (0/1/0)	[29]
		**Fallow deer** **(*Dama dama*)**	5	1/2/2	0.33 (0.33)	0.0% (0/1/4)	0.0% (0/1/4)	0.0% (0/1/4)	0.0% (0/1/4)		0.0% (0/1/4)	0.0% (0/1/4)	0.0% (0/1/4)	0.0% (0/1/4)	0.0% (0/1/4)	0.0% (0/1/4)	0.0% (0/1/4)	0.0% (0/1/4)	0.0% (0/1/4)	[8,29]
		**Elk** **(*Cervus canadiensis*)**	6	2/0/4	0.42 (0.42)	NA (0/0/6)	NA (0/0/6)	NA (0/0/6)	NA (0/0/6)		NA (0/0/6)	NA (0/0/6)	NA (0/0/6)	NA (0/0/6)	NA (0/0/6)	NA (0/0/6)	NA (0/0/6)	NA (0/0/6)	NA (0/0/6)	[8]
		**Red deer** **(*Cervus elaphus*)**	13	0/3/10	0.49 (0.33–1.67)	NA (0/0/13)	NA (0/0/13)	NA (0/0/13)	NA (0/0/13)		NA (0/0/13)	NA (0/0/13)	NA (0/0/13)	NA (0/0/13)	NA (0/0/13)	NA (0/0/13)	NA (0/0/13)	NA (0/0/13)	NA (0/0/13)	[8]
		**Red deer × Elk** **(*Cervus elaphus* × *Cervus canadiensis*)**	2	1/0/1	0.42 (0.42)	NA (0/0/2)	NA (0/0/2)	NA (0/0/2)	NA (0/0/2)		NA (0/0/2)	NA (0/0/2)	NA (0/0/2)	NA (0/0/2)	NA (0/0/2)	NA (0/0/2)	NA (0/0/2)	NA (0/0/2)	NA (0/0/2)	[8]
		**Southern pudu** **(*Pudu puda*)**	1	0/0/1	NA	0.0% (0/1/0)	0.0% (0/1/0)	**100.0%** **(1/0/0)**	0.0% (0/1/0)		0.0% (0/1/0)	0.0% (0/1/0)	0.0% (0/1/0)	0.0% (0/1/0)	0.0% (0/1/0)	0.0% (0/1/0)	0.0% (0/1/0)	0.0% (0/1/0)	0.0% (0/1/0)	[74]
Carnivora	Felidae	**African lion** **(*Panthera leo*)**	3	2/1/0	16 (14–17)	0.0% (0/2/1)	0.0% (0/2/1)	0.0% (0/2/1)	0.0% (0/2/1)		0.0% (0/2/1)	0.0% (0/2/1)	0.0% (0/2/1)	0.0% (0/2/1)	0.0% (0/2/1)	**50.0%** **(1/1/1)**	0.0% (0/2/1)	0.0% (0/2/1)	**50.0%** **(1/1/1)**	[45]
		**Cougar** **(*Felis concolor*)**	1	1/0/0	14 (14)	0.0% (0/1/0)	0.0% (0/1/0)	0.0% (0/1/0)	0.0% (0/1/0)		0.0% (0/1/0)	0.0% (0/1/0)	0.0% (0/1/0)	0.0% (0/1/0)	0.0% (0/1/0)	0.0% (0/1/0)	0.0% (0/1/0)	0.0% (0/1/0)	0.0% (0/1/0)	[44]
Chiroptera	Phyllostomidae	**Seba’s short-tailed bat** **(*Carollia perspicillata*)**	2	1/1/0	NA	0.0% (0/2/0)	0.0% (0/2/0)	**50.0%** **(1/1/0)**	0.0% (0/2/0)		0.0% (0/2/0)	**50.0%** **(1/1/0)**	**50.0%** **(1/1/0)**	0.0% (0/2/0)	0.0% (0/2/0)	0.0% (0/2/0)	0.0% (0/2/0)	0.0% (0/2/0)	0.0% (0/2/0)	[16]
	Pteropodidae	**Egyptian rousette bat *** **(*Rousettus aegyptiacus*)**	92	17/13/62	NA	**47.6%** **(20/22/50**)	0.0% (0/42/50)	0.0% (0/42/50)	0.0% (0/42/50)		0.0% (0/42/50)	0.0% (0/42/50)	0.0% (0/42/50)	0.0% (0/42/50)	0.0% (0/42/50)	0.0% (0/42/50)	0.0% (0/42/50)	0.0% (0/42/50)	0.0% (0/42/50)	[31,47]
Hystricomorpha	Cuniculidae	**Paca** **(*Cuniculus paca*)**	1	0/1/0	2 (2)	0.0% (0/1/0)	**100.0%** **(1/0/0)**	**100.0%** **(1/0/0)**	**100.0%** **(1/0/0)**		0.0% (0/1/0)	0.0% (0/1/0)	0.0% (0/1/0)	0.0% (0/1/0)	0.0% (0/1/0)	0.0% (0/1/0)	100.0% (1/0/0)	0.0% (0/1/0)	0.0% (0/1/0)	[67]
Macroscelidea	Macroscelididae	**Short-eared elephant shrew** **(*Macroscelides proboscideus*)**	1	0/0/1	NA	0.0% (0/1/0)	0.0% (0/1/0)	0.0% (0/1/0)	0.0% (0/1/0)		0.0% (0/1/0)	0.0% (0/1/0)	0.0% (0/1/0)	0.0% (0/1/0)	0.0% (0/1/0)	0.0% (0/1/0)	0.0% (0/1/0)	0.0% (0/1/0)	0.0% (0/1/0)	[6]
Primates	Atelidae	**Brown-headed spider monkey** **(*Ateles fusciceps*)**	2	0/0/2	NA	0.0% (0/2/0)	0.0% (0/2/0)	0.0% (0/2/0)	0.0% (0/2/0)		0.0% (0/2/0)	0.0% (0/2/0)	0.0% (0/2/0)	0.0% (0/2/0)	0.0% (0/2/0)	0.0% (0/2/0)	0.0% (0/2/0)	0.0% (0/2/0)	0.0% (0/2/0)	[6]
	Cebidae	**Squirrel monkey** **(*Saimiri* sp.)**	13	0/0/12	NA	0.0% (0/8/4)	0.0% (0/8/4)	0.0% (0/8/4)	**100.0%** **(8/0/4)**		0.0% (0/8/4)	0.0% (0/8/4)	0.0% (0/8/4)	0.0% (0/8/4)	**50.0%** **(4/4/4**)	0.0% (0/8/4)	0.0% (0/8/4)	0.0% (0/8/4)	**50.0%** **(4/4/4)**	[7]
		**Bolivian squirrel monkey** **(*Saimiri boliviensis*)**	1	1/0/0	<1 (<1)	0.0% (0/1/0)	0.0% (0/1/0)	**100.0%** **(1/0/0)**	0.0% (0/1/0)		**100.0%** **(1/0/0)**	0.0% (0/1/0)	0.0% (0/1/0)	0.0% (0/1/0)	0.0% (0/1/0)	0.0% (0/1/0)	0.0% (0/1/0)	0.0% (0/1/0)	0.0% (0/1/0)	[10]
		**Silvery marmoset** **(*Mico argentatus*)**	5	2/2/1	NA	0.0% (0/5/0)	0.0% (0/5/0)	**20.0%** **(1/4/0)**	**20.0%** **(1/4/0)**		0.0% (0/5/0)	0.0% (0/5/0)	0.0% (0/5/0)	0.0% (0/5/0)	0.0% (0/5/0)	0.0% (0/5/0)	0.0% (0/5/0)	**60.0%** **(3/2/0)**	0.0% (0/5/0)	[29]
	Pitheciidae	**White-faced saki** **(*Pithecia pithecia*)**	1	0/1/0	NA	0.0% (0/1/0)	0.0% (0/1/0)	0.0% (0/1/0)	0.0% (0/1/0)		0.0% (0/1/0)	0.0% (0/1/0)	0.0% (0/1/0)	0.0% (0/1/0)	0.0% (0/1/0)	0.0% (0/1/0)	0.0% (0/1/0)	0.0% (0/1/0)	0.0% (0/1/0)	[29]
Rodentia	Caviidae	**Chacoan mara** **(*Dolichotis salinicola*)**	1	0/0/1	NA	0.0% (0/1/0)	0.0% (0/1/0)	**100.0%** **(1/0/0)**	0.0% (0/1/0)		0.0% (0/1/0)	0.0% (0/1/0)	0.0% (0/1/0)	0.0% (0/1/0)	0.0% (0/1/0)	0.0% (0/1/0)	0.0% (0/1/0)	0.0% (0/1/0)	0.0% (0/1/0)	[6]
		**Patagonian mara** **(*Dolichotis patagonum*)**	2	0/0/2	NA	0.0% (0/2/0)	0.0% (0/2/0)	0.0% (0/2/0)	0.0% (0/2/0)		0.0% (0/2/0)	0.0% (0/2/0)	0.0% (0/2/0)	0.0% (0/2/0)	0.0% (0/2/0)	0.0% (0/2/0)	0.0% (0/2/0)	0.0% (0/2/0)	0.0% (0/2/0)	[6]
	Hydrochaeridae	**Capybara** **(*Hydrochoerus hydrochaeris*)**	9	6/1/2	0.28 (0.25–0.33)	**40.0%** **(2/3/4)**	**60.0%** **(3/2/4)**	**60.0%** **(3/2/4)**	0.0% (0/5/4)		0.0% (0/5/4)	0.0% (0/5/4)	0.0% (0/5/4)	**60.0%** **(3/2/4)**	0.0% (0/5/4)	0.0% (0/5/4)	0.0% (0/5/4)	0.0% (0/5/4)	0.0% (0/5/4)	[32,65]
	Sciuridae	**Japanese squirrel** **(*Sciurus lis*)**	1	0/1/0	2 (2)	**100.0%** **(1/0/0)**	**100.0%** **(1/0/0)**	**100.0%** **(1/0/0)**	0.0% (0/1/0)		0.0% (0/1/0)	0.0% (0/1/0)	0.0% (0/1/0)	0.0% (0/1/0)	0.0% (0/1/0)	0.0% (0/1/0)	0.0% (0/1/0)	0.0% (0/1/0)	0.0% (0/1/0)	[7]
		**Total**	**170**	**39/30/101**		**11.4%** **(5/39/126)**	**11.4%** **(5/39/126)**	**38.6%** **(17/27/126)**	**25.0%** **(11/33/126)**		**2.3%** **(1/43/126)**	**4.5%** **(2/42/126)**	**4.5%** **(2/42/126)**	**6.8%** **(3/41/126)**	**9.1%** **(4/40/126)**	**2.3%** **(1/43/126)**	**2.3%** **(1/43/126)**	**6.8%** **(3/41/126)**	**11.4%** **(5/39/126)**	

Both percentages and absolute numbers of cases in which gross lesions are described are presented. Reports older than 1995 were excluded from the analysis. NA is used to indicate that the data are not available for this species. * In Egyptian rousette bats, only 20.7% (19/92) of animals died from *Y. pseudotuberculosis* infection; the other animals were euthanized [43,76]. The occurrence of post-mortem lesions in this species should be viewed in light of the fact that it is unlikely that all animals were afflicted by yersiniosis at the time of death. In order to reduce bias, data on this species are considered as NA in in the ‘Total’ row.

**Table A4:** An overview of gross post-mortem observations as reported in the referred literature in birds that died from *Y. pseudotuberculosis* in zoological institutions.

								Respiratory System					Lymphoid Tissue		Liver		Gastrointestinal System			Kidneys	Circulatory System		
Order	Family	Species	Number of Animals (*n*)	Sex (M/F/NA)	Age (Years) Avg. (Range)	Body Condition (Good/Poor/NA)	Coelomic Effusion (Y/N/NA)	White-Yellow Nodules on Lungs (Y/N/NA)	Nodules on Air Sacs (Y/N/NA)	Air Sac Effusion (Y/N/NA)	Pulmonary Hyperemia (Y/N/NA)	Pulmonary Edema (Y/N/NA)	Splenomegaly (Y/N/NA)	White-Yellow Nodules on Spleen (Y/N/NA)	White or Yellow Foci on Liver (Y/N/NA)	Hepatomegaly (Y/N/NA)	Gastritis, Enteritis, Colitis, or Combination (Y/N/NA)	GI Tract Wall Edema (Y/N/NA)	Enlarged Cecal Tonsils (Y/N/NA)	White-Yellow Nodules on Kidneys (Y/N/NA)	White-Yellow Nodules on Pericardium (Y/N/NA)	**Systemic Venous Congestion** **(Y/N/NA)**	**Reference**
Bucerotiformes	Bucerotidae	**Sulawesi hornbill** **(*Rhabdotorrhinus exarhatus*)**	1	1/0/0	NA	0/1/0	0.0% (0/1/0)	0.0% (0/1/0)	0.0% (0/1/0)	0.0% (0/1/0)	0.0% (0/1/0)	0.0% (0/1/0)	**100.0%** **(1/0/0)**	0.0% (0/1/0)	0.0% (0/1/0)	**100.0%** **(1/0/0)**	0.0% (0/1/0)	0.0% (0/1/0)	0.0% (0/1/0)	0.0% (0/1/0)	0.0% (0/1/0)	0.0% (0/1/0)	[42]
		**Tarictic hornbill** **(*Penelopides panini*)**	3	3/0/0	NA	2/0/1	0.0% (0/3/0)	0.0% (0/3/0)	**33.3%** **(1/2/0)**	**33.3%** **(1/2/0)**	0.0% (0/3/0)	0.0% (0/3/0)	**66.7%** **(2/1/0)**	0.0% (0/3/0)	**66.7%** **(2/1/0)**	0.0% (0/3/0)	0.0% (0/3/0)	0.0% (0/3/0)	0.0% (0/3/0)	0.0% (0/3/0)	0.0% (0/3/0)	0.0% (0/3/0)	[42]
		**Von der Decken’s hornbill** **(*Tockus deckeni*)**	1	1/0/0	NA	0/1/0	0.0% (0/1/0)	0.0% (0/1/0)	0.0% (0/1/0)	0.0% (0/1/0)	0.0% (0/1/0)	0.0% (0/1/0)	0.0% (0/1/0)	**100.0%** **(1/0/0)**	**100.0%** **(1/0/0)**	0.0% (0/1/0)	0.0% (0/1/0)	0.0% (0/1/0)	0.0% (0/1/0)	**100.0%** **(1/0/0)**	0.0% (0/1/0)	0.0% (0/1/0)	[7]
Columbiformes	Columbidae	**Eurasian collared dove** **(*Streptopelia decaocto*)**	4	0/2/2	NA	2/0/2	0.0% (0/2/2)	0.0% (0/2/2)	0.0% (0/2/2)	0.0% (0/2/2)	**100.0%** **(2/0/2)**	**100.0%** **(2/0/2)**	0.0% (0/2/2)	**100.0%** **(2/0/2)**	**100.0%** **(2/0/2)**	0.0% (0/2/2)	**50.0%** **(1/1/2)**	0.0% (0/2/2)	0.0% (0/2/2)	0.0% (0/2/2)	0.0% (0/2/2)	0.0% (0/2/2)	[7]
		**New Zealand wood pigeon** **(*Hemiphaga novaeseelandiae*)**	3	0/0/3	NA	0/0/3	0.0% (0/3/0)	0.0% (0/3/0)	0.0% (0/3/0)	0.0% (0/3/0)	0.0% (0/3/0)	0.0% (0/3/0)	**100.0%** **(3/0/0)**	**100.0%** **(3/0/0)**	**100.0%** **(3/0/0)**	**100.0%** **(3/0/0)**	0.0% (0/3/0)	0.0% (0/3/0)	0.0% (0/3/0)	0.0% (0/3/0)	0.0% (0/3/0)	0.0% (0/3/0)	[29]
Musophagiformes	Musophagidae	**Fischer’s turaco** **(*Tauraco fischeri*)**	2	1/1/0	NA	2/0/0	**50.0%** **(1/1/0)**	0.0% (0/2/0)	**50.0%** **(1/1/0)**	0.0% (0/2/0)	0.0% (0/2/0)	0.0% (0/2/0)	**100.0%** **(2/0/0)**	0.0% (0/2/0)	**50.0%** **(1/1/0)**	**50.0%** **(1/1/0)**	0.0% (0/2/0)	**100.0%** **(2/0/0**)	0.0% (0/2/0)	0.0% (0/2/0)	0.0% (0/2/0)	0.0% (0/2/0)	[5]
Passeriformes	Estrildidae	**Zebra finch** **(*Poephila guttata*)**	2	0/0/2	NA	0/0/2	0.0% (0/2/0)	0.0% (0/2/0)	0.0% (0/2/0)	0.0% (0/2/0)	0.0% (0/2/0)	0.0% (0/2/0)	**50.0%** **(1/1/0)**	0.0% (0/2/0)	0.0% (0/2/0)	**50.0%** **(1/1/0)**	**100.0%** **(2/0/0)**	0.0% (0/2/0)	**100.0%** **(2/0/0)**	0.0% (0/2/0)	0.0% (0/2/0)	0.0% (0/2/0)	[42]
	Fringillidae	**Canary** **(*Serinus canaria*)**	6	0/0/6	NA	0/0/6	0.0% (0/6/0)	0.0% (0/6/0)	0.0% (0/6/0)	0.0% (0/6/0)	0.0% (0/6/0)	0.0% (0/6/0)	**83.3%** **(5/1/0)**	**16.7%** **(1/5/0)**	**33.3%** **(2/4/0)**	**83.3%** **(5/1/0)**	**100.0%** **(6/0/0)**	0.0% (0/6/0)	**100.0%** **(6/0/0)**	0.0% (0/6/0)	0.0% (0/6/0)	0.0% (0/6/0)	[5]
Piciformes	Ramphastidae	**Chestnut-mandibled toucan** **(*Ramphastos swainsonii*)**	1	0/1/0	NA	1/0/0	0.0% (0/1/0)	0.0% (0/1/0)	0.0% (0/1/0)	0.0% (0/1/0)	0.0% (0/1/0)	0.0% (0/1/0)	**100.0%** **(1/0/0)**	**100.0%** **(1/0/0)**	**100.0%** **(1/0/0)**	**100.0%** **(1/0/0)**	**100.0%** **(1/0/0)**	0.0% (0/1/0)	0.0% (0/1/0)	0.0% (0/1/0)	0.0% (0/1/0)	**100.0%** **(1/0/0)**	[42]
		**Keel-billed toucan** **(*Ramphastos sulfuratus*)**	2	1/1/0	NA	2/0/0	0.0% (0/2/0)	0.0% (0/2/0)	0.0% (0/2/0)	0.0% (0/2/0)	0.0% (0/2/0)	0.0% (0/2/0)	**100.0%** **(2/0/0)**	**100.0%** **(2/0/0)**	**100.0%** **(2/0/0)**	**100.0%** **(2/0/0)**	**100.0%** **(2/0/0)**	0.0% (0/2/0)	0.0% (0/2/0)	**50.0%** **(1/1/0)**	0.0% (0/2/0)	**100.0%** **(2/0/0)**	[42]
		**Toco toucan** **(*Ramphastos toco*)**	1	0/1/0	NA	0/1/0	0.0% (0/1/0)	0.0% (0/1/0)	0.0% (0/1/0)	0.0% (0/1/0)	0.0% (0/1/0)	0.0% (0/1/0)	**100.0%** **(1/0/0)**	0.0% (0/1/0)	0.0% (0/1/0)	**100.0%** **(1/0/0)**	0.0% (0/1/0)	0.0% (0/1/0)	0.0% (0/1/0)	0.0% (0/1/0)	0.0% (0/1/0)	0.0% (0/1/0)	[42]
Psittaciformes	Psittacidae	**Blue-fronted amazon** **(*Amazona aestiva*)**	1	0/1/0	7 (7)	0/1/0	0.0% (0/1/0)	**100.0%** **(1/0/0)**	0.0% (0/1/0)	0.0% (0/1/0)	0.0% (0/1/0)	0.0% (0/1/0)	0.0% (0/1/0)	**100.0%** **(1/0/0)**	**100.0%** **(1/0/0)**	0.0% (0/1/0)	0.0% (0/1/0)	0.0% (0/1/0)	0.0% (0/1/0)	**100.0%** **(1/0/0)**	**100.0%** **(1/0/0)**	0.0% (0/1/0)	[42]
		**Kaka** **(*Nestor meriondalis*)**	1	0/0/1	NA	0/0/1	0.0% (0/1/0)	0.0% (0/1/0)	0.0% (0/1/0)	0.0% (0/1/0)	0.0% (0/1/0)	0.0% (0/1/0)	**100.0%** **(1/0/0)**	**100.0%** **(1/0/0)**	**100.0%** **(1/0/0)**	**100.0%** **(1/0/0)**	0.0% (0/1/0)	0.0% (0/1/0)	0.0% (0/1/0)	0.0% (0/1/0)	0.0% (0/1/0)	0.0% (0/1/0)	[7]
		**Yellow-headed amazon** **(*Amazona oratrix*)**	1	0/1/0	0.67 (0.67)	0/1/0	0.0% (0/1/0)	**100.0%** **(1/0/0)**	0.0% (0/1/0)	0.0% (0/1/0)	0.0% (0/1/0)	0.0% (0/1/0)	0.0% (0/1/0)	**100.0%** **(1/0/0)**	**100.0%** **(1/0/0)**	0.0% (0/1/0)	0.0% (0/1/0)	0.0% (0/1/0)	0.0% (0/1/0)	**100.0%** **(1/0/0)**	**100.0%** **(1/0/0)**	0.0% (0/1/0)	[7]
	Psittaculidae	**Budgerigar** **(*Melopsittacus undulatus*)**	1	0/0/1	NA	0/0/1	0.0% (0/1/0)	0.0% (0/1/0)	0.0% (0/1/0)	0.0% (0/1/0)	0.0% (0/1/0)	0.0% (0/1/0)	**100.0%** **(1/0/0)**	**100.0%** **(1/0/0)**	**100.0%** **(1/0/0)**	**100.0%** **(1/0/0)**	0.0% (0/1/0)	0.0% (0/1/0)	0.0% (0/1/0)	0.0% (0/1/0)	0.0% (0/1/0)	0.0% (0/1/0)	[29]
		**Rainbow lorikeet** **(*Trichoglossus mollucanus*)**	1	0/0/1	NA	0/0/1	0.0% (0/1/0)	0.0% (0/1/0)	0.0% (0/1/0)	0.0% (0/1/0)	0.0% (0/1/0)	0.0% (0/1/0)	**100.0%** **(1/0/0)**	**100.0%** **(1/0/0)**	**100.0%** **(1/0/0)**	**100.0%** **(1/0/0)**	0.0% (0/1/0)	0.0% (0/1/0)	0.0% (0/1/0)	0.0% (0/1/0)	0.0% (0/1/0)	0.0% (0/1/0)	[5]
		**Total**	**41**	**7/8/16**		**9/5/17**	**3.4%** **(1/28/2)**	**6.9%** **(2/27/2)**	**6.9%** **(2/27/2)**	**3.4%** **(1/28/2)**	**6.9%** **(2/27/2)**	**6.9%** **(2/27/2)**	**72.4%** **(21/8/2)**	**51.7%** **(15/14/2)**	**65.5%** **(19/10/2)**	**62.1%** **(18/11/2)**	**41.4%** **(12/17/2)**	**6.9%** **(2/27/2)**	**27.6%** **(8/21/2)**	**13.8%** **(4/25/2)**	**6.9%** **(2/27/2)**	**10.3%** **(3/26/2)**	

Both percentages and absolute numbers of cases in which gross lesions are described are presented. Reports older than 1995 were excluded from the analysis. NA is used to indicate that the data are not available for this species.
